# Caloric vestibular stimulation: interaction between somatosensory system and vestibular apparatus

**DOI:** 10.3389/fnint.2013.00066

**Published:** 2013-09-17

**Authors:** Gabriella Bottini, Martina Gandola, Anna Sedda, Elisa R. Ferrè

**Affiliations:** ^1^Department of Brain and Behavioral Sciences, University of PaviaPavia, Italy; ^2^Cognitive Neuropsychology Center, Niguarda Ca' Granda HospitalMilan, Italy; ^3^Institute of Cognitive Neuroscience, University College LondonLondon, UK

**Keywords:** caloric vestibular stimulation, touch, neglect, multisensory integration, hemianaesthesia

Spatial and bodily representations are multisensory processes that imply the integration of several afferent signals into a coherent internal model of our egocentric space. Crucially, this model involves also the vestibular information from the balance organs in the inner ear (Ventre et al., 1984). Accordingly, vestibular system projections have been proven to overlap with the somatosensory system and with brain regions involved in body and space representation (Bottini et al., 1994, 1995; Fasold et al., 2002). These representations can be altered by a brain lesion and dramatically restored by physiological manipulations targeting specific sensory components, such as the caloric vestibular stimulation (CVS; see for a review Rossetti and Rode, 2002). CVS consists in a water irrigation of the external auditory canal, which induces a change in the temperature that leads to convection currents in the semicircular canals. This evokes a slow-phase nystagmus toward the stimulated ear and it elicits sensations of virtual body rotations and vertigo (Bárány, 1906; Silberpfennig, 1941; Bárány, 1967).

CVS has been used to modulate a wide range of cognitive and sensory functions in brain-damaged patients and in healthy participants (Utz et al., 2011). For instance, in right brain-damaged patients, CVS produces a temporary recovery of visuo-spatial neglect and associated symptoms, such as representational and personal neglect, anosognosia, somatoparaphrenia and motor neglect (see reviews in Rossetti and Rode, 2002; Kerkhoff and Schenk, 2012). Additionally, CVS also influences tactile perception: cold CVS delivered on the left ear transiently reduces tactile imperception (hemianesthesia) in both right and left brain-damaged patients (Vallar et al., 1990, 1993; Bottini et al., 2005). By contrast, the reversed stimulation (i.e., right ear cold CVS) is ineffective in left brain-damaged patients with the interesting exception of left brain-damaged patients with right visuo-spatial neglect (Vallar et al., 1993). More recently, similar cross-modal modulations have been described in healthy participants (Ferrè et al., 2010, 2011, 2012, 2013).

Various hypotheses have been suggested to explain the CVS-induced modulation on tactile perception. In particular, one of the most controversial issues in the classical and current literature concerns the *specificity* of these effects. Does CVS directly affect the somatosensory processing? Are the observed effects mediated by non-specific factors, such as ocular movements, spatial attention or general arousal?

Since Rubens (1985), most of the scientists believed that positive (e.g., deficits reduction) or negative (e.g., deficits worsening) effects of CVS on spatial deficits in neurological patients can be explained by low-level visuo-vestibular interactions reflecting the direction of the nystagmus (Rubens, 1985). When a leftward nystagmus is present, for instance during left-cold CVS or right-warm CVS, there is a positive effect. Conversely, with a rightward nystagmus (left-warm CVS and right-cold CVS) a deficits worsening is observed (Rubens, 1985; Vallar et al., 1990). However, this traditional explanation has been challenged by several clinical reports which highlighted an effective CVS-induced modulation on deficits that do not require visual control such as personal neglect (Cappa et al., 1987), anosognosia and somatoparaphrenia (Cappa et al., 1987; Bisiach et al., 1991; Rode et al., 1992). Similarly, the remission of hemianesthesia in blind-folded patients (Vallar et al., 1990) rules out this low-level interpretation.

Conversely, the role of non-specific effects such as spatial attention is still a matter of debate. This hypothesis argues that CVS may induce a reorientation of spatial attention toward the hemispace ipsilateral to the stimulated ear. Strong evidence against this hypothesis derives from a recent study on brain-damaged patients (Bottini et al., 2005) demonstrating that left-cold CVS also ameliorates right hemianesthesia in left brain-damaged patients (i.e., CVS at same water temperature, same stimulated ear and same leftwards slow-phase nystagmus), independently from the side of stimulation. These behavioral observations have been combined with neuroimaging data to identify the neurofunctional basis of CVS effects on touch perception in a group of normal participants and in one left brain-damaged patient. In this patient, we found that the remission of right hemianesthesia after cold-left CVS was associated with neural activity in the secondary somatosensory cortex (SII) of the undamaged hemisphere. The same region was bilaterally activated in healthy volunteers while they were touched on their right and left hand. Interestingly, the activation of SII for ipsilateral stimuli was of a greater extent in the right than in the left hemisphere in case of left tactile stimulation. These observations have been interpreted as a modulation that did not depend on a lower-level lateral cueing effect, but rather on the activation of the hemisphere that contains a more complete representation of the tactile and body space, the right hemisphere (Bottini et al., 2005). The involvement of SII clearly indicates an overlap between tactile and vestibular projections in the human brain (case RF; Bottini et al., 1995), and it makes explanations in terms of pure spatial effects improbable.

More recent behavioral and electrophysiological studies, in healthy participants, have strengthened this suggestion. There are at least three main crucial observations ruling out interpretation in terms of non-specific attentional effects. First, left-cold CVS affects the perception of distinct somatosensory sub-modalities, i.e., touch and pain, for both the ipsilateral and contralateral hand (Ferrè et al., 2011, 2013). A simple change in the level of spatial attention would have induced a predominant effect on the hand ipsilateral to the stimulated ear. Second, CVS differentially affects touch and pain. Indeed, while CVS increased sensitivity to tactile stimuli, it reduced levels of pain (Ferrè et al., 2013). These further observations cannot be attributed merely to a spatial attention orientation effect, as in this case we would expect the same modulatory effect in both sub-modalities. Finally, CVS enhanced the N80 wave of the somatosensory-evoked potentials (SEPs) elicited by electrical stimulation of tactile afferents (Ferrè et al., 2012). Interestingly, the N80 wave is generated in the parietal operculum (Jung et al., 2009; Eickhoff et al., 2010), a region receiving strong vestibular projections. Taken together, clinical observations and psychophysical studies give support to the notion of powerful cross-modal interactions between vestibular and somatosensory systems.

Previous studies exploring more widely CVS effects also support the idea that spatial attention does not have a pivotal role. Rorden et al. (2001) did not find an effect of left-cold CVS on covert visual attention in healthy subjects. Furthermore, cold-water bilateral CVS (simultaneous stimulations of the right and left ear) was ineffective on visual neglect in brain-damaged patients, suggesting that CVS might improve neglect through a vestibular-induced specific effect (Rode et al., 2002). Moreover, it has been suggested that CVS can also modify the internal representation of the body (see for an extensive review Lopez et al., 2008). These well documented effects have been explained by the anatomical overlap and interactions of vestibular cortex and somatosensory networks subserving elementary and more structured perceptions concerning the body representation (Lopez et al., 2008, 2012).

To conclude, this evidence suggests that in healthy volunteers the effects of CVS are *specific* and related to the activation of cortico-subcortical networks (Lopez et al., 2012) involved in cross-modal interactions between somatosensory and vestibular signals. We propose that future studies are necessary to extend these findings in neurological patients to better detail the neurophysiological interaction between the somatosensory and the vestibular systems.

## References

[B1] Bárány R. (1906). Untersuchungen über den vom vestibularapparatdes ohres reflektorisch ausgelösten rhythmischen nystagmus und seine begleiterscheinungen. Mschr. Ohrenheilk. 40, 193–297

[B2] Bárány R. (1967). “Some new methods for functional testing of the vestibular apparatus and the cerebellum. Nobel Lecture, September 11, 1916,” in Nobel Lectures Including Presentation of Speeches and Laureates' Biographies, Physiology or Medicine 1901–1921 (Amsterdam: Elsevier Publishing Company), 500–511

[B3] Bisiach E. Rusconi M. L. Vallar G. (1991). Remission of somatoparaphrenic delusion through vestibular stimulation. Neuropsychologia 29, 1029–1031 10.1016/0028-3932(91)90066-H 1762671

[B4] Bottini G. Paulesu E. Gandola M. Loffredo S. Scarpa P. Sterzi R. (2005). Left caloric vestibular stimulation ameliorates right hemianesthesia. Neurology 65, 1278–1283 10.1212/01.wnl.0000182398.14088.e8 16247057

[B5] Bottini G. Paulesu E. Sterzi R. Warburton E. Wise R. J. Vallar G. (1995). Modulation of conscious experience by peripheral sensory stimuli. Nature 376, 778–781 10.1038/376778a0 7651537

[B6] Bottini G. Sterzi R. Paulesu E. Vallar G. Cappa S. F. Erminio F. (1994). Identification of the central vestibular projections in man: a positron emission tomography activation study. Exp. Brain Res. 99, 164–169 10.1007/BF00241421 7925790

[B7] Cappa S. Sterzi R. Vallar G. Bisiach E. (1987). Remission of hemineglect and anosognosia during vestibular stimulation. Neuropsychologia 25, 775–782 10.1016/0028-3932(87)90115-1 3501552

[B8] Eickhoff S. B. Jbabdi S. Caspers S. Laird A. R. Fox P. T. Zilles K. (2010). Anatomical and functional connectivity of cytoarchitectonic areas within the human parietal operculum. J. Neurosci. 30, 6409–6421 10.1523/JNEUROSCI.5664-09.2010 20445067 PMC4791040

[B9] Fasold O. Von Brevern M. Kuhberg M. Ploner C. J. Villringer A. Lempert T. (2002). Human vestibular cortex as identified with caloric stimulation in functional magnetic resonance imaging. Neuroimage 17, 1384–1393 10.1006/nimg.2002.1241 12414278

[B10] Ferrè E. R. Bottini G. Haggard P. (2011). Vestibular modulation of somatosensory perception. Eur. J. Neurosci. 34, 1337–1344 10.1111/j.1460-9568.2011.07859.x 21978189

[B11] Ferrè E. R. Bottini G. Haggard P. (2012). Vestibular inputs modulate somatosensory cortical processing. Brain Struct. Funct. 217, 859–864 10.1007/s00429-012-0404-7 22466455

[B12] Ferrè E. R. Bottini G. Iannetti G. D. Haggard P. (2013). The balance of feelings: vestibular modulation of bodily sensations. Cortex 49, 748–758 10.1016/j.cortex.2012.01.012 22385524

[B13] Ferrè E. R. Sedda A. Gandola M. Bottini G. (2010). How the vestibular system modulates tactile perception in normal subjects: a behavioural and physiological study. Exp. Brain Res. 208, 29–38 10.1007/s00221-010-2450-9 20972670

[B14] Jung P. Baumgartner U. Stoeter P. Treede R. D. (2009). Structural and functional asymmetry in the human parietal opercular cortex. J. Neurophysiol. 101, 3246–3257 10.1152/jn.91264.2008 19357343 PMC3817274

[B15] Kerkhoff G. Schenk T. (2012). Rehabilitation of neglect: an update. Neuropsychologia 50, 1072–1079 10.1016/j.neuropsychologia.2012.01.024 22306520

[B16] Lopez C. Blanke O. Mast F. W. (2012). The human vestibular cortex revealed by coordinate-based activation likelihood estimation meta-analysis. Neuroscience 212, 159–179 10.1016/j.neuroscience.2012.03.028 22516007

[B17] Lopez C. Halje P. Blanke O. (2008). Body ownership and embodiment: vestibular and multisensory mechanisms. Neurophysiol. Clin. 38, 149–161 10.1016/j.neucli.2007.12.006 18539248

[B18] Rode G. Charles N. Perenin M. T. Vighetto A. Trillet M. Aimard G. (1992). Partial remission of hemiplegia and somatoparaphrenia through vestibular stimulation in a case of unilateral neglect. Cortex 28, 203–208 10.1016/S0010-9452(13)80048-2 1499306

[B19] Rode G. Tilikete C. Luaute J. Rossetti Y. Vighetto A. Boisson D. (2002). Bilateral vestibular stimulation does not improve visual hemineglect. Neuropsychologia 40, 1104–1106 10.1016/S0028-3932(01)00187-7 11900761

[B20] Rorden C. Karnath H. O. Driver J. (2001). Do neck-proprioceptive and caloric-vestibular stimulation influence covert visual attention in normals, as they influence visual neglect? Neuropsychologia 39, 364–375 10.1016/S0028-3932(00)00126-3 11164875

[B21] Rossetti Y. Rode G. (2002). “Reducing spatialneglect by visual and other sensory manipulations: noncognitive (physiological) routes to the rehabilitation of a cognitive disorder,” in The Cognitive and Neural Bases of Spatial Neglect, eds Karnath H. O. Milner D. Vallar G. (New York, NY: Oxford University Press), 375–396

[B22] Rubens A. B. (1985). Caloric stimulation and unilateral visual neglect. Neurology 35, 1019–1024 10.1212/WNL.35.7.1019 4010940

[B23] Silberpfennig M. D. J. (1941). Contributions to the problem of eye movements. III. Disturbances of ocular movements with pseudohemianopsia in frontal lobe tumors. Confin. Neurol. 42, 1–13 10.1159/000106147

[B24] Utz K. S. Korluss K. Schmidt L. Rosenthal A. Oppenlander K. Keller I. (2011). Minor adverse effects of galvanic vestibular stimulation in persons with stroke and healthy individuals. Brain Inj. 25, 1058–1069 10.3109/02699052.2011.607789 21879800

[B25] Vallar G. Bottini G. Rusconi M. L. Sterzi R. (1993). Exploring somatosensory hemineglect by vestibular stimulation. Brain 116(Pt 1), 71–86 10.1093/brain/116.1.71 8453466

[B26] Vallar G. Sterzi R. Bottini G. Cappa S. Rusconi M. L. (1990). Temporary remission of left hemianesthesia after vestibular stimulation. A sensory neglect phenomenon. Cortex 26, 123–131 10.1016/S0010-9452(13)80078-0 2354638

[B27] Ventre J. Flandrin J. M. Jeannerod M. (1984). In search for the egocentric reference. A neurophysiological hypothesis. Neuropsychologia 22, 797–806 10.1016/0028-3932(84)90104-0 6335575

